# l-2-Nitrimino-1,3-diazepane-4-carboxylic acid

**DOI:** 10.1107/S1600536808011835

**Published:** 2008-04-30

**Authors:** Harutyun A. Karapetyan

**Affiliations:** a Molecular Structure Research Center, National Academy of Sciences RA, Azatutyan ave. 26, 375014 Yerevan, Republic of Armenia

## Abstract

The cyclic form of l-nitro­arginine, C_6_H_10_N_4_O_4_, crystallizes with two independent mol­ecules in the asymmetric unit. According to the geometrical parameters, similar in both mol­ecules, the structure corresponds to that of l-2-nitrimino-1,3-diazepane-4-carboxylic acid; there are, however, conformational differences between the independent molecules, one of them being close to a twisted chair while the other might be described as a rather flattened boat. All six active H atoms in the two molecules are involved in hydrogen bonds, two of which are intra­molecular and four inter­molecular, forming an infinite chain of mol­ecules along the *b* axis.

## Related literature

For the crystal structures of some analogs of the title compound, see: Apreyan *et al.* (2007 ▶, 2008 ▶); Karapetyan *et al.* (2007 ▶); Petrosyan *et al.* (2005 ▶). For related literature, see: Paul *et al.* (1961 ▶); Apreyan & Petrosyan (2008 ▶).
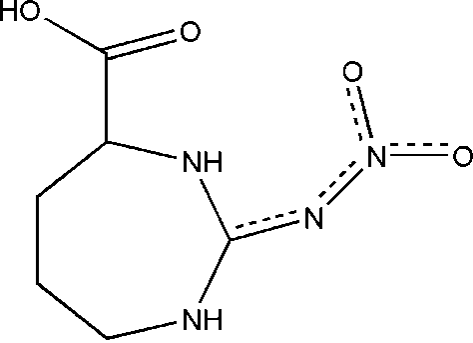


## Experimental

### 

#### Crystal data

C_6_H_10_N_4_O_4_
*M* *_r_* = 202.18
Orthorhombic, 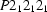

*a* = 6.9787 (14) Å
*b* = 15.233 (3) Å
*c* = 16.637 (3) Å
*V* = 1768.6 (6) Å^3^
*Z* = 8
Mo *K*α radiation
μ = 0.13 mm^−1^
*T* = 293 (2) K
0.20 × 0.17 × 0.14 mm

#### Data collection

Enraf–Nonius CAD-4 diffractometer
Absorption correction: none
13278 measured reflections
2211 independent reflections
1509 reflections with *I* > 2σ(*I*)
*R*_int_ = 0.046
3 standard reflections every 400 reflections intensity decay: none

#### Refinement

*R*[*F* ^2^ > 2σ(*F* ^2^)] = 0.041
*wR*(*F* ^2^) = 0.114
*S* = 1.06
2211 reflections
255 parameters
H-atom parameters constraned
Δρ_max_ = 0.25 e Å^−3^
Δρ_min_ = −0.21 e Å^−3^

### 

Data collection: *CAD-4 Manual* (Enraf–Nonius, 1988 ▶); cell refinement: *CAD-4 Manual*; data reduction: *HELENA* (Spek, (1997 ▶); program(s) used to solve structure: *SHELXS97* (Sheldrick, 2008 ▶); program(s) used to refine structure: *SHELXL97* (Sheldrick, 2008 ▶); molecular graphics: *SHELXTL* (Sheldrick, 2008 ▶); software used to prepare material for publication: *SHELXTL*.

## Supplementary Material

Crystal structure: contains datablocks global, I. DOI: 10.1107/S1600536808011835/bg2177sup1.cif

Structure factors: contains datablocks I. DOI: 10.1107/S1600536808011835/bg2177Isup2.hkl

Additional supplementary materials:  crystallographic information; 3D view; checkCIF report

## Figures and Tables

**Table 1:** Hydrogen-bond geometry (Å, °)

*D*—H⋯*A*	*D*—H	H⋯*A*	*D*⋯*A*	*D*—H⋯*A*
N2—H10⋯O6	0.86	2.27	2.938 (4)	134
N6—H20⋯O2^i^	0.86	2.17	2.988 (4)	158
N1—H3⋯O3	0.86	2.05	2.591 (4)	121
N5—H13⋯O7	0.86	1.92	2.571 (4)	132
O5—H11⋯N3	0.82	1.88	2.690 (4)	172
O1—H1⋯N7^ii^	0.82	1.88	2.685 (3)	169

Symmetry codes: (i) 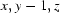; (ii) 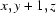.

**Fig. 1.**
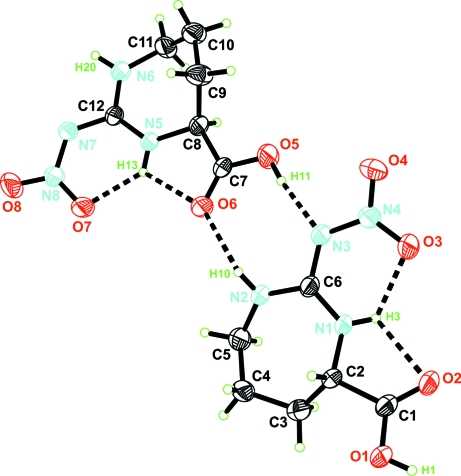
A perspective view of the crystallographically independent molecules paired via intermolecular H-bonds showing atomic numbering and displacement ellipsoids at the 50% probability.

**Fig. 2.**
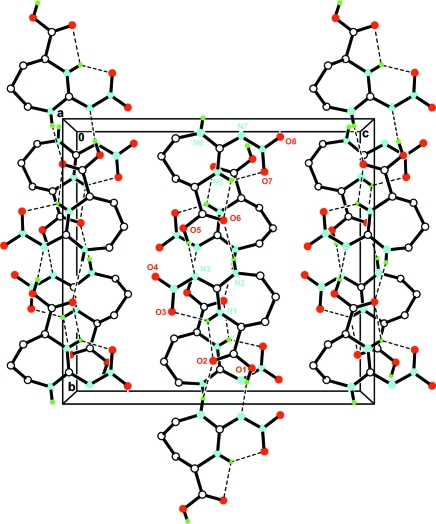
Packing of the molecules (without non-active H atoms). For clarity the non-hydrogen atoms of the crystallographically independent molecules participating in H-bonds are numbered only.

## supplementary crystallographic information

### Comment

The salts of the L-arginine have been intensively  investigated as non-linear optical materials [Petrosyan *et al.*(2005) and Karapetyan *et al.*(2007)]. Recently, reports about L-nitroarginine [Apreyan *et al.*(2008)] and its crystalline salts [Apreyan *et al.*(2007)] (a promising line of non-linear optical materials) have appeared.

We present herein a structural study of the cyclic form of L-nitroarginine, C_6_H_10_N_4_O_4_ (I), which crystallizes with two independent molecules in the unit cell. The molecule was reported for the first time by (Paul *et al.*, 1961) where it was suggested to be 2-nitro-4-carboxy-1,3- -diazacycloheptane, on the basis of chemical properties and IR spectra. According to the present single-crystal X-ray diffraction results the L-2-nitrimino-1,3-diazepane-4-carboxylic acid (L-NIDCA) form is suggested instead. A view of the H-bonded pair of crystallographically independent molecules is shown in Fig. 1. The values of bond distances and angles are in agreement with common accepted values which lead  to the proposed structural interpretation. In spite of the metric similarities there are conformational differences between the independent moieties, one of them being close to a twist-chair while the other may be described as an essentially flattened boat. All six active H atoms in the crystal are involved in hydrogen bonding (Table 1), two of them being intra- and four inter-molecular, linking crystallographically independent units and by way of which an infinite chain of molecules along the b axis is formed (Fig. 2).

### Experimental

The obtainement of crystals of the title compound consisted of a two step process.First of all, the potassium salt of (I) was obtained by the reaction of L-nitroarginine with KOH. Afterwards, by the interaction of this potassium salt with HBF4 and further separation of the poorly soluble KBF4 salt, single crystals of (I) were obtained by slow evaporation at room temperature. The compound obtained is more correctly named L-2-nitrimino-1,3-diazepane-4-carboxylic acid (L-NIDCA). Details of the obtainment of L-NIDCA and L-NIDCA^.^H_2_O, as well as vibrational spectra, thermal properties and SHG will be reported soon separately [Apreyan and Petrosyan, 2008].

### Refinement

In spite of a pronounced centrosymmetric statistics of intensities, non-centrosymmetric P2(1)2(1)2(1) was chosen as the space group, on the basis of second harmonic generation. The statistics was latter justified by the structure resolution, which presents a strong pseudo centrosymmetric character. All the H atoms were placed in geometrically calculated positions and included in the refinement in a riding model approximation, with *U*_iso_(H): 1.5*U*_eq_(of hydroxyl O atoms) and 1.2*U*_eq_ (other carrier atoms). The positional as well as anisotropic thermal parameters of non-hydrogen atoms were refined without restraints. In the absense of any significant anomalous effect, Friedel pairs were merged.

### Crystal data

**Table:**

C_6_H_10_N_4_O_4_	*F*_000_ = 848
*M**_r_* = 202.18	*D*_x_ = 1.519 Mg m^−^^3^
Orthorhombic, *P*2_1_2_1_2_1_	Mo *K*α radiation λ = 0.71073 Å
Hall symbol: P 2ac 2ab	Cell parameters from 24 reflections
*a* = 6.9787 (14) Å	θ = 14–16º
*b* = 15.233 (3) Å	µ = 0.13 mm^−^^1^
*c* = 16.637 (3) Å	*T* = 293 (2) K
*V* = 1768.6 (6) Å^3^	Block, colourless
*Z* = 8	0.20 × 0.17 × 0.14 mm

### Data collection

**Table:**

Enraf–Nonius CAD-4 diffractometer	*R*_int_ = 0.046
Radiation source: fine-focus sealed tube	θ_max_ = 27.0º
Monochromator: graphite	θ_min_ = 2.5º
*T* = 293(2) K	*h* = −7→8
ω/2θ scans	*k* = −19→19
Absorption correction: none	*l* = −21→21
13278 measured reflections	3 standard reflections
2211 independent reflections	every 400 reflections
1509 reflections with *I* > 2σ(*I*)	intensity decay: none

### Refinement

**Table:**

Refinement on *F*^2^	Secondary atom site location: difference Fourier map
Least-squares matrix: full	Hydrogen site location: inferred from neighbouring sites
*R*[*F*^2^ > 2σ(*F*^2^)] = 0.041	H-atom parameters constrained
*wR*(*F*^2^) = 0.114	*w* = 1/[σ^2^(*F*_o_^2^) + (0.0543*P*)^2^ + 0.5039*P*] where *P* = (*F*_o_^2^ + 2*F*_c_^2^)/3
*S* = 1.06	(Δ/σ)_max_ = 0.001
2211 reflections	Δρ_max_ = 0.25 e Å^−^^3^
255 parameters	Δρ_min_ = −0.21 e Å^−^^3^
Primary atom site location: structure-invariant direct methods	

### Special details

**Table:**

Geometry. All e.s.d.'s (except the e.s.d. in the dihedral angle between two l.s. planes) are estimated using the full covariance matrix. The cell e.s.d.'s are taken into account individually in the estimation of e.s.d.'s in distances, angles and torsion angles; correlations between e.s.d.'s in cell parameters are only used when they are defined by crystal symmetry. An approximate (isotropic) treatment of cell e.s.d.'s is used for estimating e.s.d.'s involving l.s. planes.
Refinement. Refinement of *F*^2^ against ALL reflections. The weighted *R*-factor *wR* and goodness of fit *S* are based on *F*^2^, conventional *R*-factors *R* are based on *F*, with *F* set to zero for negative *F*^2^. The threshold expression of *F*^2^ > σ(*F*^2^) is used only for calculating *R*-factors(gt) *etc*. and is not relevant to the choice of reflections for refinement. *R*-factors based on *F*^2^ are statistically about twice as large as those based on *F*, and *R*- factors based on ALL data will be even larger.

### Fractional atomic coordinates and isotropic or equivalent isotropic displacement parameters (Å^2^)

**Table:**

	*x*	*y*	*z*	*U*_iso_*/*U*_eq_	
O1	0.7754 (5)	0.88281 (14)	0.60814 (15)	0.0563 (8)	
H1	0.7796	0.9337	0.5922	0.085*	
O2	0.8669 (4)	0.85314 (15)	0.48298 (16)	0.0472 (7)	
O3	0.8340 (4)	0.67954 (15)	0.34868 (15)	0.0530 (8)	
O4	0.7646 (6)	0.55602 (18)	0.29427 (15)	0.0723 (10)	
O5	0.8340 (6)	0.38278 (15)	0.38570 (16)	0.0659 (10)	
H11	0.8210	0.4336	0.4009	0.099*	
O6	0.8143 (4)	0.35371 (15)	0.51572 (15)	0.0442 (7)	
O7	0.8020 (5)	0.17963 (15)	0.64914 (14)	0.0525 (8)	
O8	0.7484 (6)	0.05314 (16)	0.70190 (15)	0.0632 (9)	
N1	0.8515 (4)	0.68230 (16)	0.50423 (17)	0.0333 (7)	
H3	0.9030	0.7103	0.4649	0.040*	
N2	0.8213 (5)	0.54128 (18)	0.55682 (17)	0.0392 (8)	
H10	0.7518	0.4952	0.5506	0.047*	
N3	0.8130 (5)	0.55431 (18)	0.42317 (17)	0.0384 (8)	
N4	0.8015 (5)	0.59993 (18)	0.35350 (18)	0.0426 (8)	
N5	0.8587 (4)	0.18391 (17)	0.49647 (17)	0.0366 (7)	
H13	0.8600	0.2132	0.5407	0.044*	
N6	0.8668 (5)	0.04394 (18)	0.44140 (17)	0.0393 (7)	
H20	0.8344	−0.0099	0.4491	0.047*	
N7	0.8136 (4)	0.05420 (18)	0.57390 (16)	0.0356 (7)	
N8	0.7869 (5)	0.09867 (18)	0.64405 (18)	0.0405 (7)	
C1	0.8179 (5)	0.8301 (2)	0.5493 (2)	0.0348 (9)	
C2	0.7977 (5)	0.73407 (19)	0.57448 (18)	0.0313 (8)	
H2	0.6619	0.7230	0.5854	0.038*	
C3	0.9090 (6)	0.7139 (2)	0.6508 (2)	0.0442 (9)	
H5	1.0450	0.7133	0.6388	0.053*	
H4	0.8857	0.7598	0.6900	0.053*	
C4	0.8511 (6)	0.6259 (2)	0.6862 (2)	0.0429 (9)	
H6	0.9056	0.6202	0.7396	0.052*	
H7	0.7128	0.6236	0.6912	0.052*	
C5	0.9179 (6)	0.5504 (2)	0.6348 (2)	0.0428 (9)	
H8	1.0542	0.5571	0.6253	0.051*	
H9	0.9000	0.4964	0.6649	0.051*	
C6	0.8289 (5)	0.5961 (2)	0.4948 (2)	0.0313 (8)	
C7	0.8343 (5)	0.3309 (2)	0.4473 (2)	0.0343 (8)	
C8	0.8689 (5)	0.2356 (2)	0.42267 (19)	0.0336 (8)	
H12	0.9992	0.2310	0.4011	0.040*	
C9	0.7300 (6)	0.2026 (2)	0.3592 (2)	0.0455 (9)	
H15	0.6101	0.1870	0.3849	0.055*	
H14	0.7039	0.2496	0.3214	0.055*	
C10	0.8042 (6)	0.1241 (2)	0.3135 (2)	0.0483 (10)	
H17	0.6959	0.0893	0.2955	0.058*	
H16	0.8716	0.1446	0.2661	0.058*	
C11	0.9367 (6)	0.0661 (2)	0.36141 (19)	0.0437 (9)	
H19	0.9578	0.0122	0.3317	0.052*	
H18	1.0594	0.0955	0.3667	0.052*	
C12	0.8479 (5)	0.09769 (19)	0.5036 (2)	0.0306 (8)	

### Atomic displacement parameters (Å^2^)

**Table:**

	*U*^11^	*U*^22^	*U*^33^	*U*^12^	*U*^13^	*U*^23^
O1	0.107 (2)	0.0200 (12)	0.0418 (15)	−0.0005 (15)	0.0200 (17)	−0.0027 (11)
O2	0.0777 (19)	0.0213 (11)	0.0427 (15)	−0.0003 (12)	0.0161 (14)	0.0023 (11)
O3	0.093 (2)	0.0271 (13)	0.0389 (15)	0.0039 (13)	0.0023 (15)	0.0058 (12)
O4	0.137 (3)	0.0472 (18)	0.0325 (15)	−0.009 (2)	−0.009 (2)	−0.0075 (14)
O5	0.137 (3)	0.0214 (13)	0.0387 (15)	0.0028 (16)	0.0074 (17)	0.0042 (11)
O6	0.0735 (18)	0.0234 (12)	0.0357 (13)	0.0021 (12)	0.0036 (13)	−0.0026 (11)
O7	0.096 (2)	0.0264 (12)	0.0353 (14)	−0.0023 (14)	0.0031 (15)	−0.0035 (12)
O8	0.115 (3)	0.0393 (15)	0.0347 (15)	−0.0006 (19)	0.0091 (18)	0.0087 (13)
N1	0.0498 (18)	0.0193 (13)	0.0307 (16)	−0.0017 (13)	0.0056 (14)	0.0026 (13)
N2	0.064 (2)	0.0215 (13)	0.0319 (15)	−0.0086 (13)	0.0034 (15)	0.0008 (13)
N3	0.062 (2)	0.0205 (14)	0.0325 (16)	−0.0005 (14)	0.0049 (15)	−0.0004 (13)
N4	0.067 (2)	0.0277 (14)	0.0334 (17)	0.0067 (15)	−0.0013 (17)	−0.0016 (15)
N5	0.063 (2)	0.0188 (13)	0.0278 (16)	−0.0042 (14)	0.0032 (15)	0.0005 (13)
N6	0.064 (2)	0.0198 (13)	0.0339 (16)	0.0009 (14)	0.0020 (15)	−0.0001 (13)
N7	0.0563 (19)	0.0212 (14)	0.0294 (15)	−0.0001 (13)	0.0012 (15)	−0.0011 (12)
N8	0.063 (2)	0.0258 (14)	0.0332 (17)	−0.0007 (14)	0.0005 (17)	0.0020 (14)
C1	0.042 (2)	0.0233 (18)	0.039 (2)	−0.0016 (14)	0.0035 (17)	−0.0020 (17)
C2	0.0437 (19)	0.0193 (14)	0.0310 (17)	−0.0004 (14)	0.0016 (16)	−0.0015 (13)
C3	0.063 (2)	0.0331 (16)	0.0363 (18)	−0.0035 (17)	−0.0063 (19)	−0.0031 (14)
C4	0.067 (2)	0.0336 (18)	0.0278 (18)	0.0030 (17)	−0.0058 (17)	0.0015 (15)
C5	0.061 (2)	0.0317 (17)	0.036 (2)	0.0055 (18)	−0.0003 (19)	0.0027 (15)
C6	0.037 (2)	0.0225 (16)	0.0344 (19)	−0.0017 (13)	0.0061 (15)	0.0002 (15)
C7	0.050 (2)	0.0197 (17)	0.0335 (19)	−0.0037 (14)	−0.0001 (16)	0.0014 (15)
C8	0.046 (2)	0.0205 (14)	0.0345 (18)	0.0019 (14)	0.0019 (16)	0.0002 (13)
C9	0.061 (2)	0.0350 (17)	0.0401 (19)	0.0117 (17)	−0.0133 (19)	−0.0061 (15)
C10	0.078 (3)	0.0290 (18)	0.038 (2)	0.0057 (18)	−0.006 (2)	−0.0030 (16)
C11	0.069 (3)	0.0296 (17)	0.0326 (19)	0.0080 (17)	0.0094 (19)	−0.0046 (15)
C12	0.041 (2)	0.0198 (15)	0.0309 (18)	0.0005 (13)	−0.0014 (15)	0.0023 (14)

### Geometric parameters (Å, °)

**Table:**

O1—C1	1.300 (4)	N7—N8	1.362 (4)
O1—H1	0.8200	N7—C12	1.365 (4)
O2—C1	1.207 (4)	C1—C2	1.528 (4)
O3—N4	1.236 (3)	C2—C3	1.519 (4)
O4—N4	1.219 (4)	C2—H2	0.9800
O5—C7	1.294 (4)	C3—C4	1.519 (5)
O5—H11	0.8200	C3—H5	0.9700
O6—C7	1.199 (4)	C3—H4	0.9700
O7—N8	1.241 (3)	C4—C5	1.507 (5)
O8—N8	1.216 (3)	C4—H6	0.9700
N1—C6	1.332 (4)	C4—H7	0.9700
N1—C2	1.459 (4)	C5—H8	0.9700
N1—H3	0.8600	C5—H9	0.9700
N2—C6	1.328 (4)	C7—C8	1.527 (4)
N2—C5	1.469 (4)	C8—C9	1.520 (5)
N2—H10	0.8600	C8—H12	0.9800
N3—N4	1.354 (4)	C9—C10	1.508 (5)
N3—C6	1.356 (4)	C9—H15	0.9700
N5—C12	1.321 (4)	C9—H14	0.9700
N5—C8	1.460 (4)	C10—C11	1.506 (5)
N5—H13	0.8600	C10—H17	0.9700
N6—C12	1.327 (4)	C10—H16	0.9700
N6—C11	1.457 (4)	C11—H19	0.9700
N6—H20	0.8600	C11—H18	0.9700
			
C1—O1—H1	109.5	C3—C4—H7	109.2
C7—O5—H11	109.5	H6—C4—H7	107.9
C6—N1—C2	126.6 (3)	N2—C5—C4	115.5 (3)
C6—N1—H3	116.7	N2—C5—H8	108.4
C2—N1—H3	116.7	C4—C5—H8	108.4
C6—N2—C5	127.5 (3)	N2—C5—H9	108.4
C6—N2—H10	116.3	C4—C5—H9	108.4
C5—N2—H10	116.3	H8—C5—H9	107.5
N4—N3—C6	121.1 (3)	N2—C6—N1	122.2 (3)
O4—N4—O3	121.6 (3)	N2—C6—N3	112.6 (3)
O4—N4—N3	115.0 (3)	N1—C6—N3	125.2 (3)
O3—N4—N3	123.2 (3)	O6—C7—O5	125.1 (3)
C12—N5—C8	127.9 (3)	O6—C7—C8	123.3 (3)
C12—N5—H13	116.0	O5—C7—C8	111.6 (3)
C8—N5—H13	116.0	N5—C8—C9	112.0 (3)
C12—N6—C11	127.1 (3)	N5—C8—C7	106.2 (3)
C12—N6—H20	116.5	C9—C8—C7	113.6 (3)
C11—N6—H20	116.5	N5—C8—H12	108.3
N8—N7—C12	121.1 (3)	C9—C8—H12	108.3
O8—N8—O7	122.1 (3)	C7—C8—H12	108.3
O8—N8—N7	115.1 (3)	C10—C9—C8	113.2 (3)
O7—N8—N7	122.8 (3)	C10—C9—H15	108.9
O2—C1—O1	125.0 (3)	C8—C9—H15	108.9
O2—C1—C2	123.7 (3)	C10—C9—H14	108.9
O1—C1—C2	111.4 (3)	C8—C9—H14	108.9
N1—C2—C3	115.4 (3)	H15—C9—H14	107.8
N1—C2—C1	105.9 (3)	C11—C10—C9	114.1 (3)
C3—C2—C1	112.0 (3)	C11—C10—H17	108.7
N1—C2—H2	107.7	C9—C10—H17	108.7
C3—C2—H2	107.7	C11—C10—H16	108.7
C1—C2—H2	107.7	C9—C10—H16	108.7
C4—C3—C2	111.5 (3)	H17—C10—H16	107.6
C4—C3—H5	109.3	N6—C11—C10	114.5 (3)
C2—C3—H5	109.3	N6—C11—H19	108.6
C4—C3—H4	109.3	C10—C11—H19	108.6
C2—C3—H4	109.3	N6—C11—H18	108.6
H5—C3—H4	108.0	C10—C11—H18	108.6
C5—C4—C3	111.9 (3)	H19—C11—H18	107.6
C5—C4—H6	109.2	N5—C12—N6	122.5 (3)
C3—C4—H6	109.2	N5—C12—N7	124.8 (3)
C5—C4—H7	109.2	N6—C12—N7	112.7 (3)
			
C6—N3—N4—O4	−171.5 (4)	N4—N3—C6—N2	174.4 (3)
C6—N3—N4—O3	11.6 (6)	N4—N3—C6—N1	−6.2 (5)
C12—N7—N8—O8	176.6 (4)	C12—N5—C8—C9	43.1 (5)
C12—N7—N8—O7	−3.9 (6)	C12—N5—C8—C7	167.7 (4)
C6—N1—C2—C3	65.9 (5)	O6—C7—C8—N5	3.5 (5)
C6—N1—C2—C1	−169.6 (3)	O5—C7—C8—N5	−177.9 (3)
O2—C1—C2—N1	0.9 (5)	O6—C7—C8—C9	127.1 (4)
O1—C1—C2—N1	−179.2 (3)	O5—C7—C8—C9	−54.3 (4)
O2—C1—C2—C3	127.5 (4)	N5—C8—C9—C10	−80.9 (4)
O1—C1—C2—C3	−52.6 (4)	C7—C8—C9—C10	158.7 (3)
N1—C2—C3—C4	−72.3 (4)	C8—C9—C10—C11	30.0 (5)
C1—C2—C3—C4	166.4 (3)	C12—N6—C11—C10	−69.0 (5)
C2—C3—C4—C5	69.8 (4)	C9—C10—C11—N6	47.6 (5)
C6—N2—C5—C4	66.1 (5)	C8—N5—C12—N6	8.0 (6)
C3—C4—C5—N2	−69.2 (4)	C8—N5—C12—N7	−171.0 (3)
C5—N2—C6—N1	−30.9 (6)	C11—N6—C12—N5	12.6 (6)
C5—N2—C6—N3	148.5 (3)	C11—N6—C12—N7	−168.3 (3)
C2—N1—C6—N2	−25.9 (5)	N8—N7—C12—N5	0.1 (5)
C2—N1—C6—N3	154.8 (3)	N8—N7—C12—N6	−178.9 (3)

### Hydrogen-bond geometry (Å, °)

**Table:**

*D*—H···*A*	*D*—H	H···*A*	*D*···*A*	*D*—H···*A*
N2—H10···O6i	0.86	2.27	2.938 (4)	134
N6—H20···O2i	0.86	2.17	2.988 (4)	158
N1—H3···O2i	0.86	2.21	2.629 (3)	110
N1—H3···O3i	0.86	2.05	2.591 (4)	121
N5—H13···O6i	0.86	2.20	2.625 (4)	110
N5—H13···O7i	0.86	1.92	2.571 (4)	132
O5—H11···N3i	0.82	1.88	2.690 (4)	172
O5—H11···O4i	0.82	2.60	3.084 (4)	119
O1—H1···N7i	0.82	1.88	2.685 (3)	169
O1—H1···O8i	0.82	2.59	3.033 (3)	116

Symmetry codes: i; i; i.
